# Epidemiology of atrial fibrillation and risk of CVD mortality among hypertensive population: A prospective cohort study in Northeast China

**DOI:** 10.3389/fcvm.2022.955685

**Published:** 2022-07-28

**Authors:** Zhi Du, Min Lin, Yuanmeng Tian, Li Jing, Shuang Liu, Guangxiao Li, Tong Jia, Qun Sun, Lei Shi, Jixu Sun, Wen Tian, Liying Xing

**Affiliations:** ^1^Department of Cardiology, The First Affiliated Hospital, Zhejiang University School of Medicine, Hangzhou, China; ^2^Department of Cardiovascular Medicine, Benxi Central Hospital, Benxi, China; ^3^Institute of Preventive Medicine, China Medical University, Shenyang, China; ^4^Department of Chronic Disease Preventive and Control, Liaoning Provincial Center for Disease Control and Prevention, Shenyang, China; ^5^Department of Ultrasound, The Fourth Hospital of China Medical University, Shenyang, China; ^6^Department of Medical Record Management Center, The First Affiliated Hospital of China Medical University, Shenyang, China; ^7^College of Information Science and Engineering, Northeastern University, Shenyang, China; ^8^Department of Chronic Disease, Disease Control and Prevention of Chaoyang City, Chaoyang, China; ^9^Department of Chronic Disease, Disease Control and Prevention of Liaoyang City, Liaoyang, China; ^10^Department of Chronic Disease, Disease Control and Prevention of Dandong City, Dandong, China; ^11^Department of Geriatric Cardiology, The First Affiliated Hospital of China Medical University, Shenyang, China

**Keywords:** atrial fibrillation, oral anticoagulant therapy, risk factor, CVD mortality, epidemiology

## Abstract

**Background:**

Determining risk factors of cardiovascular disease (CVD)-related mortality and evaluating their influence are important for effectively reducing corresponding mortality. However, few research findings have estimated the relationship between atrial fibrillation (AF) and CVD-related mortality among hypertension individuals.

**Objective:**

The objective of this study was to investigate the epidemiology of AF in a hypertension population and determine the relationship between AF and CVD-related mortality.

**Methods:**

Using a multistage, stratified, and cluster random sampling method, the prospective cohort study with a median follow-up of 3.51 years enrolled 10,678 hypertensive participants at baseline. The prevalence, awareness, and anticoagulation data of AF in this focal population were carefully assessed. Stepwise logistic regression and Cox regression analysis were respectively performed to evaluate the determinants of AF and the association between AF and CVD-related mortality.

**Results:**

The overall prevalence of AF was 1.3% (95% CI, 1.1%−1.6%) in the hypertensive population, and it was higher in men than in women (1.8% vs. 1.0%, respectively; p=0.001). The awareness of AF was 53.1%, and the rate of oral anticoagulant (OAC) therapy was only 4.2%, although all AF participants should have required according to the European Society of Cardiology guidelines. The determinants of AF included elder, male, and history of coronary heart disease in the hypertensive population. Besides, compared with individuals without AF, the risk of CVD-related mortality significantly increased in the hypertensive population with AF (HR 3.37, 95% CI 2.10–5.40).

**Conclusion:**

Our results indicated a huge burden of AF and underuse of OAC therapy for them in a community-based hypertensive population. Considering that most of the risk factors of AF were unmodifiable in hypertensive individuals, as well as its high risk of mortality, long-term interventions including AF education, timely screening, and widespread use of OACs should be emphasized in the focal populations.

## Introduction

Atrial fibrillation (AF), the most common cardiac arrhythmia, has been accepted as the conventional factor for a series of adverse outcomes in clinical practice, including stroke, heart failure, and mortality (1, 2). Recent data suggest that AF has affected more than 33.3 million people worldwide and resulted in 195,300 deaths in 2015 alone (3). Furthermore, the number of patients with AF is expected to increase dramatically for years to come, owing to factors, such as aging, increasing screening, and predisposing factors (4, 5). Notably, some cardiovascular diseases (CVD) and its determinants, for example, heart failure, are common coexistent conditions in patients with AF, and each status could aggravate the other (6).

Hypertension whose effect is the same as heart failure is another common comorbidity in patients with AF. Robust results from multiple cohorts have asserted that hypertension could also trigger AF, with an approximately 21.6% population-attributable fraction, and further deteriorate it (7–9). As both AF and hypertension increase the risk of CVD, especially ischemic stroke and embolism, hypertension patients with AF would have a much higher risk of adverse outcomes (10). Fortunately, the widespread use of oral anticoagulants (OAC) is an efficient prevention measure to improve the prognosis in these patients (11). Hence, it is necessary to understand the epidemiological status of AF in individuals with hypertension and further develop appropriate evidence-based and actionable management strategies to improve the prognosis of AF in these participants. However, limited studies have focused on the epidemiology of AF in hypertensive individuals. In terms of the Chinese hypertensive population, the prevalence of AF was only acquired in southern individuals (12), the unrepresentative survey with low response rates (13), a small sample population enrolled in the hospital, (14) and a highly selected population (15), as well as inadequate estimation of management (12–15). Taking into account ethnic and regional differences and the aforementioned limitations, the precise burden and management situation of AF in a community-based Chinese hypertensive population is largely unexplored.

It is well-established that determining risk factors for CVD-related mortality and evaluating their influence is meaningful for effectively reducing corresponding mortality. However, few research findings have estimated the relationship between AF and CVD-related mortality among hypertension individuals (16). In the Chinese hypertensive population, to date, the relationship between AF and CVD-related mortality has only been evaluated in elderly individuals (17). Hence, the precise risk ratio of AF increasing the risk of CVD-related mortality is unclear in community-based hypertensive populations. In this context, a prospective cohort study was carried out among a large representative population comprising hypertensive adults in northeast China, and the prevalence of AF was assessed along with awareness, anticoagulation data, relevant risk factors, as well as the relationship between AF and CVD-related mortality, to fill in the abovementioned epidemiological gaps.

## Materials and methods

### Participants

The design and enrollment process of the study have been previously described (10, 11). Briefly, using a multistage, stratified, and cluster random sampling method, this baseline survey was undertaken from September 2017 to March 2019 in Liaoning Province. Three urban districts (Zhenan, Liuerpu, and Gongchangling) and four rural counties (Liaoyang, Chaoyang, Donggang, and Lingyuan) were randomly selected. From these seven regions, 19 rural villages, and eight communities were randomly selected. The permanent residents aged more than 40 years in these regions were invited to participate after the exclusion of those who had mental health issues or were pregnant (*n* = 22,009). A total of 18,796 (85.4%) accomplished the baseline investigation. Subsequently, once in 2 years, we collected participants' death details by telephone to participants' families, grass-root doctors, and local medical institutions. Hence, we fully understand the survival status of each participant. In this study, we excluded 8,188 non-hypertension participants. Eventually, data from 10,678 hypertensive patients (4,310 men and 6,368 women) were analyzed. The study was approved by the Central Ethics Committee at the China National Center for Cardiovascular Disease. Written informed consent was obtained from all study participants.

### Questionnaire and measurements

At baseline, data on demographic information, lifestyle, comorbidities, and medical and drug histories were collected through face-to-face interviews by a team comprising specialists trained in the prevention and control of chronic disease, cardiologists, and neurologists, who used standardized self-administered questionnaires during the single clinic visit. The trained staff scanned the questionnaire and manually extracted the relevant information with quality inspection and double entry to ensure that the data were recorded truthfully and carefully, with an accuracy rate of an overwhelming 98%.

Physical measurements including weight, height, and waist circumference (WC) of patients without shoes and wearing lightweight clothing were measured to the nearest 0.1 kg and 0.1 cm, respectively. The body mass index (BMI) was calculated as the patient's weight (kg) divided by the square of the height (m^2^). Using a standardized automatic electronic sphygmomanometer (J30; Omron, Kyoto, Japan), blood pressure (BP) was measured three times after at least 5-min rest intervals. All participants were advised to avoid caffeinated beverages and strenuous exercise for at least 30 min before the BP measurements. During the measurement, the participants were instructed to position their upper arms at their heart level. The average of the three recorded BP measurements was calculated and used in the analyses. Twelve-lead ECGs (resting, 10 s) were performed on each participant through a MAC 5500 System (GE Healthcare; Little Chalfont, UK). All ECG traces were analyzed manually by at least two well-trained cardiologists with the assistance of a magnifier and calipers.

Fasting blood samples were collected using BD Vacutainer tubes containing ethylenediaminetetraacetic acid (EDTA; Becton, Dickinson and Co., Franklin Lakes, NJ, USA) from participants after at least 8 h of overnight fasting. Serum samples were isolated from whole blood, stored at −20°C, and subsequently analyzed on an Abbott Diagnostics C800i auto-analyzer (Abbott Laboratories, Abbott Park, IL, USA) using commercial kits. Fasting blood glucose (FBG); glycosylated hemoglobin (HbA1c); serum lipid profiles including total cholesterol (TC), triglyceride (TG), high-density lipoprotein cholesterol (HDL-C), and low-density lipoprotein cholesterol (LDL-C); and other biochemical indicators were measured. In each laboratory, 10% of specimens were rechecked by China's Ministry of Health's National Center for Clinical Laboratory to ensure test accuracy.

### Definition

Hypertension was defined as a mean systolic BP (SBP) ≥140 mmHg or a mean diastolic BP (DBP) ≥90 mmHg, and/or self-reported use of antihypertensive medication in the past 2 weeks (18) The ECG-based diagnosis of AF was confirmed by at least two independent cardiologists. Participants who were previously identified with AF by a physician based on ECG findings were also recognized as patients with AF (15) Current smoking was identified as smoking ≥ 1 cigarette a day for at least 12 months. Current drinking was defined as drinking at least once a week. Regular exercise was defined as moderate-intensity exercise, equivalent to walking, at least 3 times per week and for at least 30 min at a time. Overweight/obesity was defined as a BMI ≥25 kg/m^2^ according to WHO criteria (11) Central obesity was determined as WC ≥90 cm in men and WC ≥80 cm in women. Diabetes mellitus was defined as an FBG ≥7.0 mmol/L or HbA1c ≥6.5%, and/or self-reported diagnosis that was previously determined by a physician (19) Dyslipidemia was diagnosed if subjects met one or more of the following criteria: (1) high TC, serum TC ≥6.22 mmol/L; (2) high TG, serum TG ≥2.26 mmol/L; (3) high LDL, serum LDL-C ≥4.14 mmol/L; (4) low HDL-C, serum HDL-C <1.04 mmol/L; or (5) self-reported use of lipid-regulating medications over the previous 4 weeks (20) Awareness of the disease was identified if participants answered “Yes” to the question, “Have you ever been diagnosed with a specific disease (such as AF, hypertension, diabetes, dyslipidemia, and CHD) by a certified doctor?” CHD was defined as angina requiring hospitalization, myocardial infarction, or any revascularization procedure.

### Judgment of clinical outcomes

During the follow-up visits, we collected their relevant information through face-to-face interviews, and telephone to participants' families, grass-root doctors, and local medical institutions. Participants' medical records and death certificates were collected in detail from local medical institutions, grass-root doctors, and family members of participants. An endpoint assessment committee comprising specialists, trained cardiologists, and neurologists reviewed these materials and made a final decision. The CVD-related mortality was determined as deaths caused by ischemic heart disease, heart failure, arrhythmia, stroke, and pulmonary edema (21). If the survival participants conducted follow-up visits and face-to-face interviews, the last interview was the follow-up time. Otherwise, we collected participants' relevant information by telephone to participants' families, grass-root doctors, and local medical institutions. The follow-up time was the time of death. If they survived, but not attended face-to-face interviews, the follow-up time was defined as the time of telephone follow-up.

### Statistical analysis

Continuous variables were reported as means and standard deviations, and categorical variables were presented as frequencies and percentages. Student's *t*-test and chi-square tests were, respectively, performed to compare intergroup differences. The direct age- and sex-standardized method was implemented to calculate the standardized prevalence of AF according to the Sixth China Population Census data. The univariate and stepwise multivariate logistic regressions were used to determine the risk factors of AF. Only variables with statistical significance in univariate analysis, including gender, age, lack of exercise, high TG, and history of CHD, were included in stepwise regression analysis. The Kaplan-Meier curves were performed to explore the cumulative incidence of CVD-related mortality, and the intergroup difference was evaluated using the log-rank test. Stepwise multivariable Cox proportional hazard models were performed to determine the association between AF and CVD-related mortality, after adjusting for age, area, sex, education level, current smoking, lack of exercise, overweight/obesity, central obesity, and history of stroke. The corresponding 95% confidence intervals (CIs) were calculated, and *p*-values <0.05 were considered statistically significant. All statistical analyses were performed using the SPSS software version 22.0 (SPSS Inc., Chicago, IL, USA).

## Results

### Baseline characteristics

This study included 10,678 participants (mean age: 62.5 ± 9.7 years, male participants: 40.4%, urban participants: 25.9%). Clinical characteristics by region and sex are shown in Table 1. Participants from rural regions had significantly lower age, BMI, WC, HbA1c, TG, and LDL-C, and higher mean SBP and DBP, and HDL-C than those from urban regions (all *p* < 0.001). Meanwhile, a higher proportion of low education, low income, current smoking, current drinking, and lack of exercise were also observed in rural regions (all *p* < 0.001). Besides, there was a significant difference between male and female subjects for all clinical characteristics, including age, BMI, mean SBP and DBP, HbA1c, TG, TC, LDL-C, HDL-C, level of education and income, current smoking and drinking, history of CHD, and lack of exercise (all *p* < 0.05).

**Table 1:** Characteristics of the study sample by region and sex.

**Characteristics**	**Total**	**Region**		**Sex**		***p*** **value for region**	***p*** **value for sex**
		**Urban**	**Rural**	**Male**	**Female**		
Participants, n (%)	10,678 (100)	2762 (25.9)	7916 (74.1)	4310 (40.4)	6368 (59.6)		
Mean age, years	62.5 ± 9.7	63.1 ± 9.2	62.3 ± 9.8	62.8 ± 10.1	62.3 ± 9.4	<0.001	0.008
Education, %							
Primary school or lower	55.0	28.9	64.1	45.7	61.3	<0.001	<0.001
Middle school	34.3	51.3	28.4	39.7	30.7		
High school or above	10.7	19.8	7.5	14.6	8.0		
Income, RMB %							
<5,000	38.5	7.6	49.3	34.7	41.1	<0.001	<0.001
5,000–19,999	33.9	23.6	37.5	33.3	34.2		
≥20,000	27.6	68.8	13.2	32.0	24.7		
Current smoking, %	23.4	19.2	24.9	49.1	6	<0.001	<0.001
Current drinking, %	26.5	21.9	28.1	54.3	7.6	<0.001	<0.001
Lack of exercise, %	15.6	9.3	17.8	13.7	17.0	<0.001	<0.001
Mean BMI, kg/m^2^	25.3 ± 3.8	25.6 ± 3.4	25.2 ± 3.9	24.9 ± 3.6	25.6 ± 3.8	<0.001	<0.001
Overweight/obesity, %	62.7	67.5	61.0	58.4	65.6	<0.001	<0.001
WC, cm	85.4 ± 12.4	86.5 ± 15.9	85.0 ± 11.0	86.1 ± 14.7	84.9 ± 10.6	<0.001	<0.001
Central obesity, %	56.4	62.7	54.2	35.8	70.4	<0.001	<0.001
Mean SBP, mmHg	157.2 ± 18.7	151.5 ± 16.6	159.1 ± 18.9	156.5 ± 18.0	157.6 ± 19.1	<0.001	0.002
Mean DBP, mmHg	91.3 ± 10.9	89.4 ± 10.1	92.0 ± 11.1	93.0 ± 10.6	90.2 ± 10.9	<0.001	<0.001
FPG, mmol/L	6.3 ± 2.0	6.3 ± 2.2	6.3 ± 2.0	6.3 ± 1.9	6.4 ± 2.1	0.072	0.062
HbA1c, %	5.7 ± 1.1	5.8 ± 1.2	5.6 ± 1.0	5.6 ± 1.0	5.8 ± 1.1	<0.001	<0.001
Diabetes mellitus, %	21.5	26.6	19.8	19.7	22.8	<0.001	<0.001
TG, mmol/L	1.8 ± 1.6	2.0 ± 1.9	1.8 ± 1.6	1.8 ± 1.8	1.9 ± 1.5	<0.001	<0.001
High TG, %	21.0	24.4	19.8	18.9	22.5	<0.001	<0.001
TC, mmol/L	5.2 ± 1.2	5.2 ± 1.3	5.2 ± 1.1	5.0 ± 1.2	5.4 ± 1.1	0.495	<0.001
High TC, %	16.6	17.1	16.4	11.6	19.9	0.410	<0.001
LDL-C, mmol/L	2.6 ± 1.0	3.0 ± 0.9	2.5 ± 1.0	2.5 ± 0.9	2.7 ± 1.0	<0.001	<0.001
High LDL, %	6.5	10.7	5.1	4.1	8.2	<0.001	<0.001
HDL-C, mmol/L	1.8 ± 0.7	1.3 ± 0.4	1.9 ± 0.7	1.7 ± 0.7	1.8 ± 0.7	<0.001	<0.001
Low HDL, %	11.5	30.3	5.0	13.1	10.5	<0.001	<0.001
Dyslipidemia, %	40.0	55.0	34.8	36.2	42.7	<0.001	<0.001
CHD, %	6.5	8.7	5.7	5.5	7.2	<0.001	0.001

*Data are presented as mean ± SD or %*.

*P-value for region: p values between urban and rural areas. P value for sex: p values between male and female*.

*BMI, body mass index; CHD, coronary heart disease; DBP, diastolic blood pressure; FBG, fasting blood glucose; HDL-C, high-density lipoprotein cholesterol; LDL-C, low-density lipoprotein cholesterol; SBP, systolic blood pressure; TG, triglycerides; TC, total cholesterol; WC, waist circumference*.

### AF prevalence

Of the 10,678 subjects, 143 had AF, and the overall crude prevalence of AF was 1.3% (95% CI, 1.1–1.6%). The prevalence rate increased with age, from 0.2% among adults aged 40–49 years to 4.9% among adults aged ≥80 years, and it was significantly higher among male than female participants (1.8 vs. 1.0%, *p* = 0.001). Among men, the prevalence of AF in the population aged 40–49 years was just 0.4%, which increased sharply to 1.6% in those aged 60–69 years and 5.3% in those aged ≥80 years. Although the prevalence of AF was higher among urban residents than among rural residents, there was no significant statistical difference (1.6 vs. 1.3%, *p* = 0.248) (Table 2).

**Table 2:** Prevalence of AF by region and sex among the study participants.

**Age group (years)**	**No of AF (total number)**	**Region**		**Sex**		**Total**	***p*** **value for region**	***p*** **value for sex**
		**Urban**	**Rural**	**Male**	**Female**			
40–49	2 (1,067)	1.0 (0.4–2.5)	0	0.4 (0.2–1.0)	0	0.2 (0.1–0.4)	0.033	0.200
50–59	20 (2,881)	0.5 (0.0–0.1)	0.8 (0.4–1.1)	1.1 (0.5–1.8)	0.4 (0.1–0.7)	0.7 (0.4–1.0)	0.505	0.033
60–69	54 (4,228)	1.6 (0.9–2.4)	1.1 (0.8–1.5)	1.6 (1.0–2.2)	1.1 (0.7–1.5)	1.3 (0.9–1.6)	0.202	0.109
70–79	47 (2,092)	2.4 (1.1–3.7)	2.2 (1.5–2.9)	2.9 (1.8–4.0)	1.8 (1.0–2.5)	2.2 (1.6–2.9)	0.788	0.100
≥80	20 (410)	5.1 (0.6–9.5)	4.8 (2.4–7.3)	5.3 (2.0–8.6)	4.5 (1.7–7.3)	4.9 (2.7–7.0)	1.000	0.720
Overall	143 (10,678)	1.6 (1.1–2.0)	1.3 (1.0–1.5)	1.8 (1.4–2.2)	1.0 (0.8–1.3)	1.3 (1.1–1.6)	0.248	0.001
ASSR		1.3	0.8	1.2	0.7	0.9		

*Percentages represent the number of subjects with AF/total population (%). AF, atrial fibrillation; ASSR, age- and sex- standardized rate*.

### Awareness and oral anticoagulant (OAC) therapy of AF

**Figure 1:**
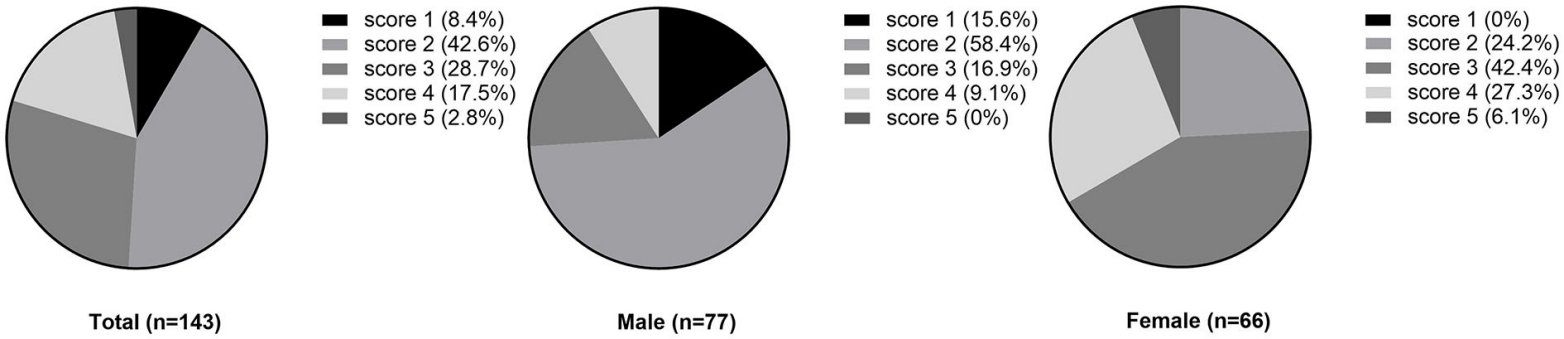
The CHA2DS2-VASc score in hypertensive population with atrial fibrillation.

**Figure 2:**
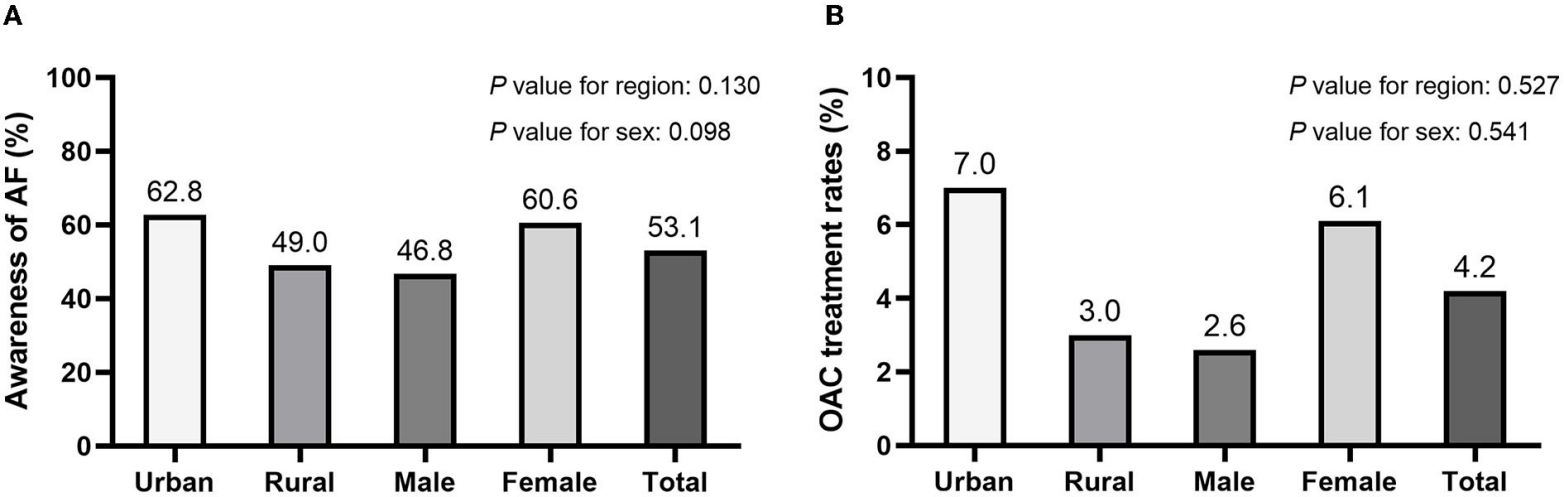
The awareness **(A)** and oral anticoagulant (OAC) usage **(B)** of AF among hypertensive patients.

The overall awareness of AF was 53.1% in this investigation population. No statistically significant difference was observed between region and sex in awareness of AF (urban vs. rural: 62.8 vs. 49.0%, *p*=0.130; male vs. female: 46.8 vs. 60.6%, *p* = 0.098, respectively). According to the CHA2DS2-VASc risk score (mean score: 2.6), all hypertension patients with AF required oral anticoagulant (OAC) therapy. However, the overall OAC therapy rate was only 4.2%. Of the six participants taking OAC, 3 received oral warfarin and the remaining three received an appropriate dosage of oral DOAC. Compared with rural areas, the proportion of OAC therapy in urban areas was higher without statistical significance (7.0 vs. 3.0%, respectively, *p* = 0.527). Similarly, a higher rate of OAC therapy was noted among female than male participants (2.6 vs. 6.1%, respectively, *p* = 0.541) (Figures 1, 2).

### Risk factors of AF

A univariate and stepwise multivariate logistic regression was performed to determine the risk factors of AF. According to the results of multivariate logistic regression, the age, gender, and history of CHD were significantly independent variables associated with AF. Specifically, the risk of AF was significantly higher in men than in women, increasing by approximately 76% (OR 1.76, 95% CI: 1.26–2.47). Meanwhile, for every 10 year increase in age, the risk of AF increased by 92% (OR: 1.92, 95% CI: 1.60–2.30). Besides, we also found that the history of CHD was an independent risk factor for AF with an OR of 5.60 (95% CI: 3.85–8.14). While lack of exercise and high TG were significantly associated with AF in univariate regression analysis, there was no significant statistical difference in subsequent stepwise multivariate logistic regression (Table 3).

**Table 3:** The risk factors associated with AF among hypertensive patients from northeast China.

**Variables**	**Univariate model**		**Multivariate model**	
	**OR (95%CI)**	***P*** **value**	**OR (95%CI)**	***P*** **value**
Male *vs*. female	1.74 (1.25–2.42)	0.001	1.76 (1.26–2.47)	0.001
Age per 10 years increase	2.03 (1.70–2.41)	<0.001	1.92 (1.60–2.30)	<0.001
Lack exercise	1.77 (1.20–2.59)	0.004	NA	-
High TG	0.57 (0.35–0.93)	0.024	NA	-
History of CHD	6.32 (4.38–9.14)	<0.001	5.60 (3.85–8.14)	<0.001

*CHD, coronary heart disease; TG, triglycerides*.

### The relationship between AF and CVD-cause mortality

**Figure 3:**
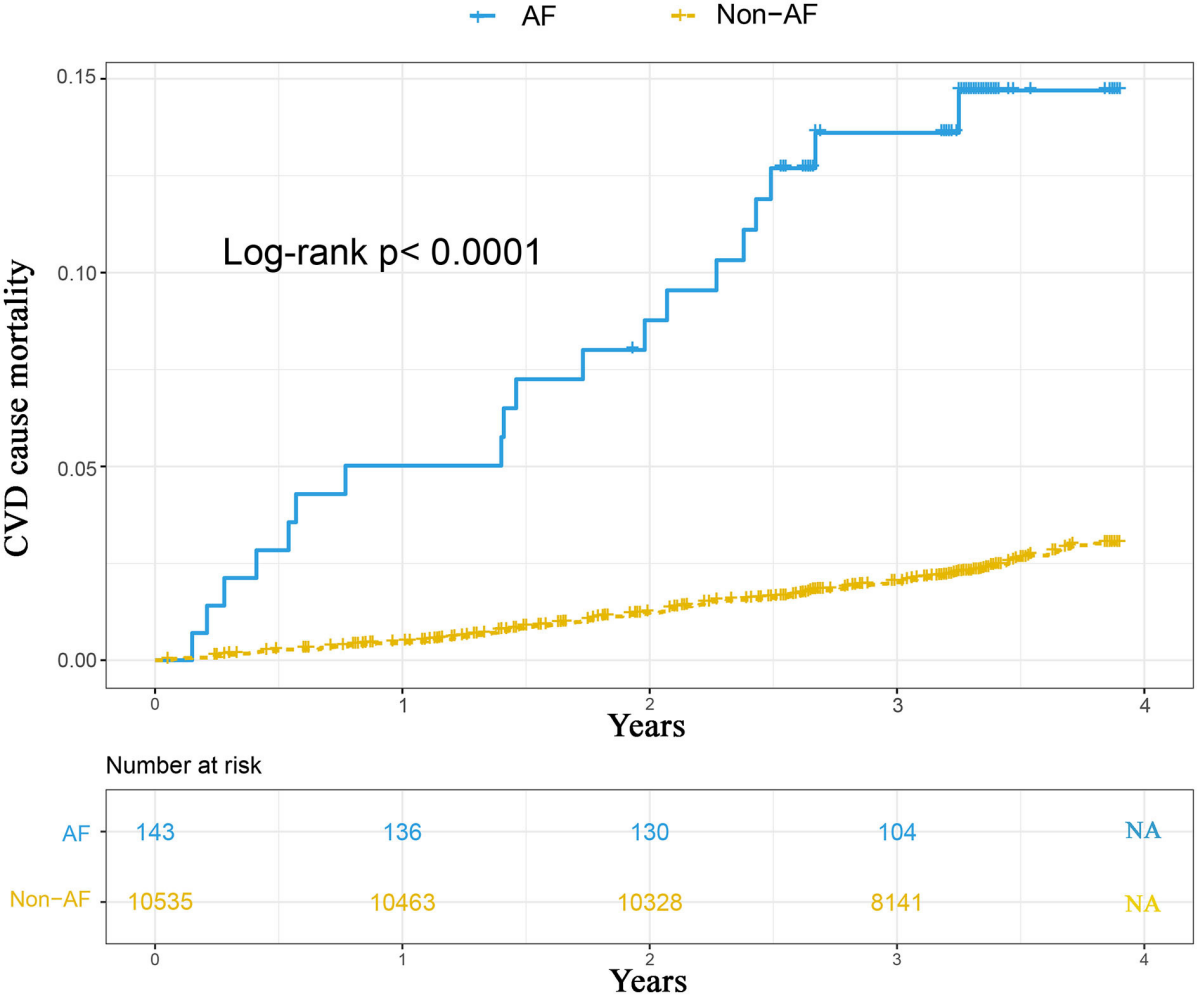
The Kaplan-Meier curves comparing the incidence of CVD mortality between hypertensive patients with AF and without AF.

**Figure 4:**
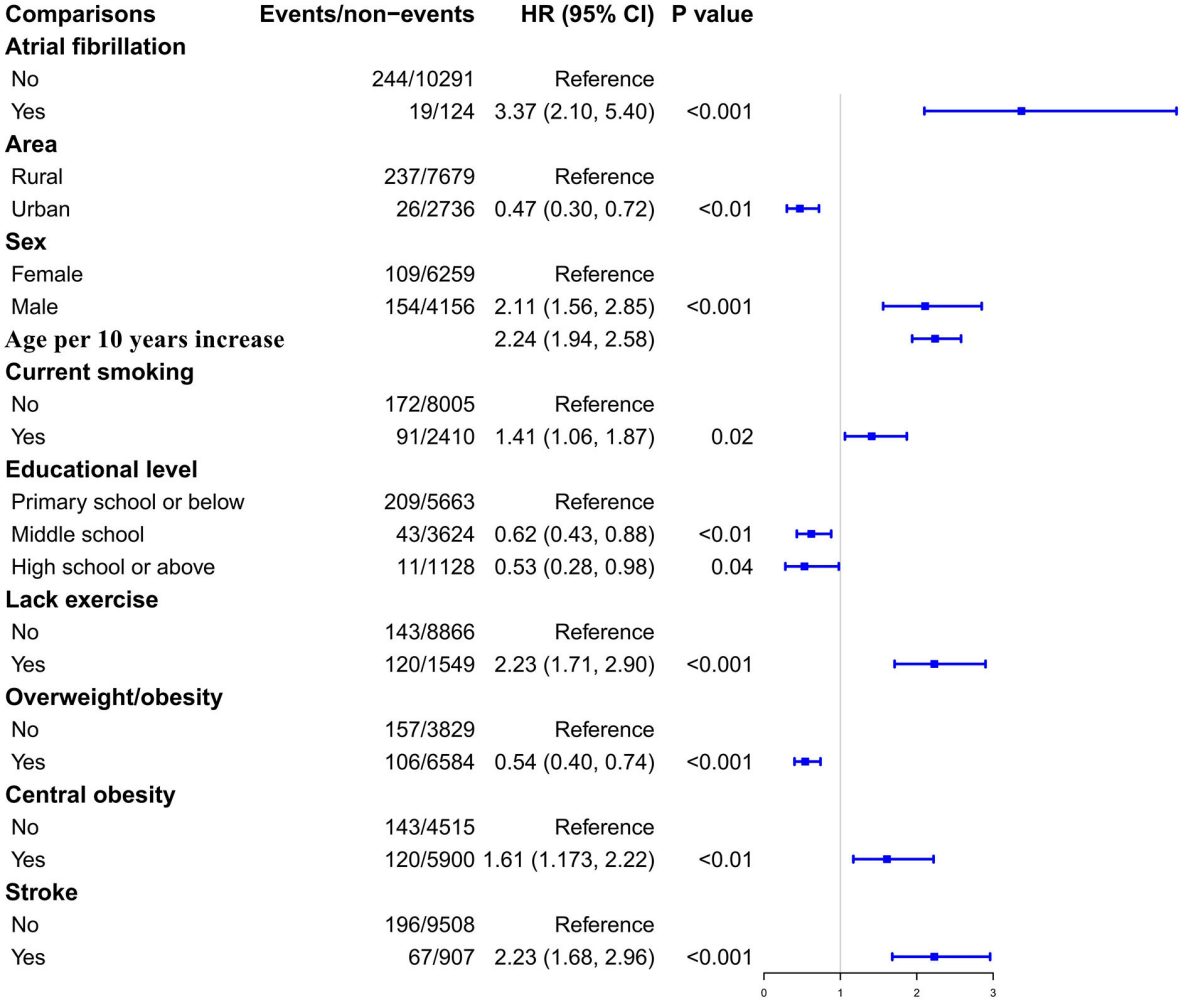
The Cox proportional hazards regression analysis exploring the association between AF and the hazard ratio of subsequent CVD mortality.

During a median follow-up of 3.51 years (interquartile range, 3.18–3.51; range 0.05–3.90), 263 participants developed CVD-cause mortality. The unadjusted Kaplan–Meier curves showed that the crude incidence rates of CVD-related mortality were significantly higher among patients with AF (log-rank *P* < 0.0001) (Figure 3). Cox proportional hazards regression analysis was used to explore the relationship between AF and CVD mortality. Ultimately, area, sex, current smoking, educational level, lack of exercise, overweight/obesity, central obesity, and stroke were reserved in stepwise Cox regression analysis. The results indicate that AF was independently associated with adverse outcomes, with an HR of 3.37 (95% CI, 2.10–5.40), after adjustment for the aforementioned conventional risk factors (Figure 4). Furthermore, in the sensitivity analysis, the association between AF and CVD-related mortality was evaluated by area stratification. The statistical difference was only observed in rural areas (Table 4). Besides, during follow-up periods, eight patients with AF developed CVD-cause mortality in the AF awareness group and 11 participants with AF developed CVD-caused mortality in the AF unawareness group. The univariate and multivariate Cox proportional hazard models were used to determine the relationship between AF awareness and CVD-cause mortality. There was no significant statistical difference in univariate Cox regression (HR, 0.622; 95% CI, 0.250–1.547). After adjusting for confounding factors, the association between AF awareness and CVD-cause mortality was not observed (HR, 0.610; 95% CI, 0.226–1.645).

**Table 4:** The relationship between AF and CVD-cause mortality evaluated by area stratification.

**Variables**	**Urban**		**Rural**	
	**HR (95%CI)**	***P*** **value**	**HR (95%CI)**	***P*** **value**
Atrial fibrillation	1.31 (0.17–9.91)	0.793	3.70 (2.27–6.01)	<0.001
Male *vs*. female	4.93 (1.85–13.12)	0.001	1.89 (1.37–2.59)	<0.001
Age per 10 years increase	2.00 (1.35–2.97)	0.001	2.27 (1.95–2.64)	<0.001
Current smoking	NA	-	1.41 (1.05–1.91)	0.023
Educational level				
Primary school or below	NA	-	Reference	
Middle school	NA	-	0.60 (0.41–0.88)	0.010
High school; or above	NA	-	0.41 (0.18–0.93)	0.032
Lack exercise	NA	-	2.27 (1.72–2.98)	<0.001
Overweight/obesity	NA	-	0.55 (0.40–0.76)	<0.001
Central obesity	NA	-	1.52 (1.08–2.12)	0.015
Stroke	NA	-	2.33 (1.74–3.13)	<0.001

## Discussion

This study carefully assessed the prevalence of AF, anticoagulation data, related risk factors, and its prognosis in a hypertensive population. The major findings of this study were as follows: (1) 1.3% prevalence of AF in the hypertensive population, which was higher in men than in women (1.8 vs. 1.0%; *p* = 0.001); However, no significant difference was found between urban and rural areas (1.6 vs. 1.3%; *p* = 0.248). (2) The awareness of AF was 53.1%, and only 4.2% actually received OAC therapy, while all AF participants were required according to the ESC guidelines (1) (3) The independent risk factors of AF included age, gender, and history of CHD. (4) Compared with hypertensive individuals without AF, the risk of CVD-related mortality increased by 37% in hypertensive patients with AF after adjustment for conventional risk factors. These findings indicate that hypertensive patients in the community had a considerable burden of AF and an unacceptable control status. Given the mostly unmodifiable risk factors and potential adverse effects of AF, early identification of hypertensive patients with AF and promotion of the usage of OACs are of great significance to improve the prognosis of this population.

### AF prevalence and its risk factors

Global studies support the findings of ethnic and regional variation in the prevalence of AF, and these differences are probably attributable to differences in study design, genetics, socioeconomic, and environmental factors (5, 22). In general, the prevalence in Europe and North America is high, while that in Asia is comparatively low (4, 5). For patients with hypertension, as mentioned above, limited studies comprehensively investigated the epidemiology of AF in this population. The Echocardiographic Heart of England Screening study, including 388 hypertensive participants, indicated the prevalence of AF was up to 3.9% in this focal UK population (23). In the Thai nationally representative survey enrolled 13,207 hypertensive patients from 831 public hospitals, AF was determined in 457 individuals, with a prevalence of 3.46% (24). However, the diagnosis of AF was limited in the two studies, only defined based on self-reported history in the first study and only based on ECG at baseline in the latter study, which inevitably led to underestimated results. Besides, because the average age of the participants was relatively young (62.5 vs. >83.5 years), the prevalence of AF in our study was lower than that in the Hypertension in the Very Elderly Trial, which included 3,273 hypertensive individuals aged ≥80 years (1.3 vs. 5.8%) (6).

In terms of Chinese hypertensive population, recent results from a nationally representative, cross-sectional study—the China Hypertension Survey—with 21,243 participants aged ≥35 years confirmed that the prevalence of AF was 1.1% in this population (13). Regrettably, this latest national survey had a relatively low response rate (62.5%), and AF was only defined based on self-reported history. It seemed reasonable that the prevalence of AF in our study was higher than the latest national data (1.3 vs. 1.1%). Meanwhile, the AF prevalence was also higher in our study than that in a retrospective cross-sectional study that enrolled 7,808 hypertensive individuals in economically developed Guangdong (1.3 vs. 1.0%) (12). As for northeast China, the prevalence of AF in our study was slightly higher than that of the general population in the same area (1.3 vs. 1.1–1.2%) (10, 15), and lower than results from rural northeast China (1.3 vs. 1.7%) (15). The positive correlation that has been observed between AF and socioeconomic status (25) likely implies the rapid urbanization of northeast rural populations with consequent lifestyle changes because our research included both rural and urban populations. Our results were consistent with this explanation because we observed that the prevalence of AF was not significantly different between the urban and rural populations (*p*=0.248).

In this study, the prevalence of AF increased with age, which was in accordance with the findings of previous studies (4, 10, 15, 26). Furthermore, we found that male subjects had a higher prevalence of AF, which is also similar to most previous studies (4, 10, 25). Notably, the subsequent identification of AF risk factors confirmed the abovementioned conclusions again in our study. Sex-related differences might be due to the differential distribution of environmental risk factors and the susceptibility of men and women to different risk factors (10, 25). In addition, we found a correlation between the history of CHD and AF, which seemed to be predictable. However, some recognized common risk factors for AF, such as diabetes and dyslipidemia, were not statistically significant in our research (27). On the one hand, it might be because the average age of participants was elder and all of them were hypertensive patients, which weakened the influence of these factors on AF. At the same time, this association was only evaluated in a cross-sectional study rather than a causal link. Overall, we found that most of the risks of AF come from inevitable causes. Therefore, early screening and effective management of AF are particularly crucial given its harmful prognosis.

### Awareness of OAC therapy for AF

This study has highlighted the suboptimal awareness and treatment of AF in patients with hypertension. Both the AHA/ACC/HRS and ESC guidelines have recommended OACs for the efficient prevention of stroke and embolism in patients with AF (28, 29). Unfortunately, OACs are underused among patients with AF in the real world (30). This phenomenon was extremely common in the general Chinese population, wherein only 1.0–4.1% of participants received OAC therapy (31), despite compelling evidence showing that anticoagulation has significant benefits for Asian patients with AF (32). Even in AF patients with ischemic stroke, the usage rate of warfarin remained as low as 19.4% in China (33). Our study observed a usage rate of 4.2% for OACs, which was slightly higher than the range of 1.0–4.1% in the general Chinese population but significantly lower than the range of 9.4–33.3% in the hospital-based population (31). Of course, the usage rate was also slightly higher than that of the general population from the same areas (10), but it was still unacceptably lower than in Europe and North America (4.2% in our study vs. 67% in Europe vs. 50% in North America) (34).

As already mentioned, our study population comprised hypertensive patients with an average age of >60 years. Therefore, an increased risk of hemorrhage may be the main reason for the underuse of OACs (5). Meanwhile, racial and regional differences in the awareness of AF, which is higher in developed countries but lower in China, might have an important impact on its treatment (35). Moreover, the lack of access to coordinated regular international normalized ratio testing and the greater cost of non-vitamin K OACs (e.g., dabigatran) are also currently major obstacles to their widespread use (33). Because the risk of stroke increases with blood pressure, the absolute benefit of OACs increases in patients with hypertension than in those without. Therefore, clinicians must realize hypertension itself should not be a reason to avoid using OACs as a preventive treatment for patients with AF.

### AF and CVD-related mortality

Our results provided strong clinical evidence that AF was an independent risk factor for CVD-related mortality in a Chinese hypertensive population. Not all previous studies have found that AF might increase the risk of mortality (6). However, these results from rare studies indicate that the correlation between AF and CVD-related mortality seems to be acceptable in hypertensive populations. In 4,736 elderly individuals with isolated systolic hypertension aged 60 years or older, the Systolic Hypertension in the Elderly Program, Vagaonescu et al. found the presence of AF would increase the risk of CVD-related mortality with an HR of 2.39 (95% CI 1.05 to 5.43) at 4.7 years of follow-up and 2.21 (1.54-3.17) at 14.3 years of follow-up, which was similar to our findings (HR 3.37, 95% CI 2.10–5.40) (16). In addition, in elderly hypertensive individuals, results from a prospective cohort study enrolled 3,922 participants aged ≥60 years confirmed the correlation between AF and CVD-related mortality again (HR 3.78, 95% CI 2.17–6.58) (17). Different from the participants in the above studies, our results claimed that this conclusion was not only appropriate in elderly hypertensive individuals but could also apply to the general hypertensive population. Furthermore, our results indicated that the association between CVD mortality and AF was only observed in rural areas. In fact, only 26 urban participants developed CVD-cause mortality during the follow-up period. The limited number of events might result in false-negative results. Besides, the relationship between AF awareness and CVD-cause mortality was not observed in this study. Of course, the limited number of AF, as well as the limited number of events because of the short follow-up time, could affect the statistical results.

### Limitations

Although this study was based on a large community-based population with an acceptable nonresponse rate (14.6%), it has some limitations. First, some paroxysmal AF cases were likely missed owing to a single ECG examination, leading to an underestimation of the prevalence of AF in our investigation. Paroxysmal AF should be diagnosed by frequent repeated ECG or ambulatory ECG monitoring, neither of which is suitable for large population surveys. Second, although our study included a large number of hypertensive patients, these participants were from northeast China and >40 years old. This may reduce the applicability of our results to other populations. Third, women and rural participants comprised a high proportion of the overall study population (59.6 and 74.1%, respectively); thus, selection bias was inevitable in the study. Nevertheless, the standardized prevalence was calculated based on the standardized population, which somewhat minimizes selection bias. Besides, because the AF was only determined at baseline, risk factors for AF were assessed as associations rather than causal links. The HAS-BLED score, the liver, and kidney function were not fully evaluated among patients with AF, although these were crucial for OAC therapy. Some confounding factors were not collected at baselines, including COPD, chronic heart failure, chronic kidney disease, thyroid disease, the duration of AF, and control of hypertension. Ultimately, although the relationship between the left atrium and AF has been accepted, its diameter was not measured at baseline. Notably, this is an exploratory analysis, and large-sample prospective studies are needed to confirm the results of this study.

## Conclusion

Leveraging the community-based survey, the huge burden and poor management of AF were carefully indicated in the hypertensive population. In view of the results that the risk factors for AF were mostly inevitable, and the risk of adverse outcomes significantly increased in hypertensive individuals with AF, the integrated measures, including AF education, timely screening, and widespread use of OACs, should be implemented in this focal population.

## Data availability statement

The raw data supporting the conclusions of this article will be made available by the authors, without undue reservation.

## Ethics statement

The studies involving human participants were reviewed and approved by the Central Ethics Committee at the China National Center for Cardiovascular Disease. The patients/participants provided their written informed consent to participate in this study. Written informed consent was obtained from the individual(s) for the publication of any potentially identifiable images or data included in this article.

## Author contributions

LX and WT were responsible for the concept and design of the study. LX was responsible for the study coordination and conduct. ZD and ML contributed to the drafting of the manuscript. GL, YT, LJ, ML, and ZD collected and analyzed the data. LX, QS, LS, JS, and TJ interpreted the data. All authors contributed to the article and approved the submitted version.

## Funding

This study was supported by the Department of Science and Technology of Liaoning Province (2018225065 and 2019JH2/10300001) and the National Natural Science Foundation of China (81701699).

## Conflict of interest

The authors declare that the research was conducted in the absence of any commercial or financial relationships that could be construed as a potential conflict of interest.

## Publisher's note

All claims expressed in this article are solely those of the authors and do not necessarily represent those of their affiliated organizations, or those of the publisher, the editors and the reviewers. Any product that may be evaluated in this article, or claim that may be made by its manufacturer, is not guaranteed or endorsed by the publisher.
